# Association and Multimodal Model of Retinal Mid-Peripheral Capillary Free Zones with Structural and Functional Parameters in Diabetic Patients Without Clinical Retinopathy

**DOI:** 10.1007/s44402-026-00041-3

**Published:** 2026-03-04

**Authors:** Ganesh B. Jonnadula, Swetha Ravichandran, Andrew Rothstein, Keisha Brown, Rohit Dhakal, Safal Khanal, Maria B. Grant, Edmund Arthur

**Affiliations:** 1 School of Optometry, University of Alabama at Birmingham, Birmingham, Alabama USA; 2 Department of Ophthalmology and Visual Sciences, Heersink School of Medicine, University of Alabama at Birmingham, Birmingham, Alabama USA

**Keywords:** Capillary free zones, Diabetic retinopathy, Electroretinography, Optical coherence tomography angiography, Retinal thickness

## Abstract

**Purpose:**

To investigate the association between mid-peripheral capillary free zones (CFZs) and retinal structural and functional metrics in diabetics without diabetic retinopathy (DR).

**Methods:**

This cross-sectional study included 45 eyes from 28 diabetics without DR and 46 eyes from 31 controls (mean age in both groups, 59 years). Macular optical coherence tomography (OCT) scans were acquired for retinal nerve fibre layer (RNFL) and ganglion cell layer thickness measurements. Thickness measurements were obtained using the Early Treatment of Diabetic Retinopathy Study grid. Retinal mid-peripheral CFZs were computed from OCT angiography images using custom MATLAB software. Retinal function was evaluated as a pilot exploratory objective using full-field flash electroretinography. Correlations between mid-peripheral CFZs and retinal structure and function were assessed using linear mixed-effect models, accounting for the association between eyes, while receiver operating characteristic curves were used to compare the multimodal models.

**Results:**

Larger periarteriole CFZs were associated with thinner inner inferior RNFL thickness (*β* = −0.48, *p* = 0.03) in diabetics without DR. Functionally, there was no significant association between the mid-peripheral CFZs and ERG parameters (*p* > 0.05) in the no DR group; these findings should be interpreted with caution given the pilot nature of the functional data. The multimodal model of vascular and structural parameters had a modestly improved area under the curve (AUC) and specificity compared to the model of vascular parameters alone (AUC = 0.85 versus 0.83, specificity = 0.65 versus 0.54, respectively).

**Conclusions:**

These findings demonstrate that enlarged mid-peripheral periarteriole CFZs are associated with thinner RNFL in diabetics without clinical retinopathy. The multimodal model of vascular and structural metrics showed modestly improved diagnostic ability. This study shows early novel retinal vascular and neural associations in diabetics without clinical retinopathy and demonstrates the potential utility of a multimodal model for discriminating this group from healthy controls.

Key Points

- Mid-peripheral capillary free zones have been identified as a potential early vascular biomarker for diabetics without clinical retinopathy.
- The study shows a significant association between these early retinal microvascular defects and neurodegeneration in diabetics without diabetic retinopathy.
- A multimodal diagnostic model, combining both vascular and structural parameters, shows enhanced ability to discriminate between normal older adults and diabetics without clinical retinopathy.

## Introduction

Diabetic retinopathy (DR) is a disabling and blinding microvascular complication of diabetes mellitus (DM) and one of the leading causes of global vision loss [1]. The public health burden of DM has risen substantially, with a 31.43% increase in prevalence between 1990 and 2022 [2]. Classical DR diagnosis has relied primarily on observable retinal vascular alterations such as perfusion abnormalities, leakage, exudates and retinal detachments. However, these clinical signs typically manifest late in the disease process, long after the initial pathological changes have occurred. Advances in retinal imaging technology have the potential for the detection of subtle changes in the neurovascular unit, necessary for early diagnosis, treatment and more sensitive monitoring of disease progression [3, 4].

Optical coherence tomography angiography (OCTA) has revolutionised the assessment of retinal microvasculature, enabling a vast amount of data describing quantitative vascular biomarkers for early DR. The two most widely studied OCTA-derived variables are vessel density and foveal avascular zone (FAZ) area [5]. However, these retinal vascular biomarkers have limitations that reduce their clinical utility in the detection of subclinical or preclinical stages of DR. Vessel density measurements, for instance, are susceptible to confounders such as image noise, differing scan quality and segmentation errors [6, 7]. Furthermore, anatomical variations such as inherent disparities in vessel calibre/diameter can impact vessel density measurements per se [8, 9], thus undermining between-person comparisons as well as the validity of this measure as a biomarker. Similarly, FAZ area measurement, even though indicative of central macular perfusion, is often insufficient as a solitary biomarker, particularly in early or mild disease [10–13]. Its changes are primarily restricted to the central macula, perhaps failing to recognise microvascular loss in the periphery of the retina, which may be a precursor to changes observed in the fovea [14]. Additionally, the size of FAZ can exhibit a ‘saturation effect’ in advanced DR, wherein its measurement may no longer rise with increasing capillary dropout. Combined with the high inter-person variability that has been documented even among healthy subjects [10–13], these factors limit the sensitivity and specificity of FAZ metrics for detecting nascent DR [15]. This highlights the need for alternative vascular biomarkers with higher sensitivity and specificity for the early detection of diabetic retinal changes.

Apart from obvious vascular changes, DR has also been described as a neurosensory disease where neural degeneration occurs before clinically apparent retinopathy [16]. Fluctuating glucose levels in DM influence both the vasculature and the structural neural retina [17]. Although the temporal pattern of vascular versus neural impairment remains under debate [18–22], early assessment of both components is critical. The photopic negative response (PhNR) and oscillatory potentials (OPs) from electroretinography (ERG) are reliable tools for evaluating retinal ganglion cell dysfunction, and PhNR has been suggested as a sensitive biomarker for early neurodegenerative changes in DR [23, 24]. However, whether early retinal vascular alterations, more specifically the mid-peripheral capillary free zones (CFZs; periarteriole and perivenule), are related to these neuronal functional changes or not is still unclear. Our earlier work has shown the presence of dilated mid-peripheral CFZs in diabetic patients without clinical retinopathy, compared to age-matched controls, and has identified them as a novel potential vascular biomarker for the identification of early DR [15]. An enlarged retinal mid-peripheral CFZ represents a larger distance that oxygen and nutrients must diffuse to reach the inner retinal neurons, specifically the retinal nerve fibre layer (RNFL) and ganglion cell layer (GCL) [15]. This may lead to potential ischaemia and a biological penalty on the structure and function of these neurons. In addition, our prior work also showed that a multimodal model of the mid-peripheral CFZs, in addition to other known vascular parameters such as the FAZ metrics and vessel density, had an improved diagnostic ability and sensitivity, but reduced specificity compared to the diagnostic performance of the mid-peripheral CFZs alone [15].

While our earlier work identified enlarged mid-peripheral CFZs in diabetics without clinical retinopathy [15], the relationship between these vascular changes and underlying neural structure and function remains unknown. Therefore, the current study was undertaken to: (1) examine the extent to which an association exists between mid-peripheral CFZ width and objective retinal structural measurements of RNFL and GCL thickness in diabetic patients without clinical retinopathy; (2) examine the extent to which an association exists between mid-peripheral CFZ width and retinal functional measurements obtained from the ERG in the same group and (3) compare the discriminative capability of a multimodal model, comprising both vascular and structural parameters, with a model comprising only vascular parameters for the discrimination of diabetics without clinical retinopathy as assessed by the Early Treatment of Diabetic Retinopathy Study (ETDRS) criteria from normal older adults. Thus, this study addresses the gap regarding how enlargement of the mid-peripheral CFZs is related to retinal structure and function in diabetic patients without clinical retinopathy, and seeks to improve upon an earlier multimodal model that included only retinal vascular parameters.

## Methods

The study was approved by the University of Alabama at Birmingham (UAB) institutional review board and adhered to the tenets of the Declaration of Helsinki. Informed consent was obtained from all participants after a comprehensive explanation of the study’s purpose and potential implications of the study.

### Study Participants

This study included 45 eyes from 28 patients with type 2 diabetes without DR (mean age, 59 years; range, 40–71 years) and 46 eyes from 31 controls without DM (mean age, 59 years; range, 46–78 years; Table 1), an identical cohort to our previously powered study [15]. While our prior work introduced the mid-peripheral CFZs in this cohort and assessed a vascular-only diagnostic model, the current study conducts a novel investigation into the association of these CFZs with detailed retinal structural (OCT) and functional (ERG) parameters. Furthermore, a new multimodal model was developed and tested that integrates these structural metrics, which was not done previously. Of these participants, 17 in the diabetic group and 15 in the control group had data included from both eyes, while 11 diabetics and 16 control participants had data from a single eye. Fellow eyes were excluded if they had suboptimal OCTA image quality (e.g., poor signal, motion artefacts) or failed to meet other study inclusion criteria (e.g., best-corrected visual acuity >0.30 logMAR, active ocular disease). These criteria were applied uniformly to both groups. The two groups were well-matched for age (*p* = 0.77) and sex (*p* = 0.76). For the diabetic participants without DR, the mean glycated haemoglobin (HbA1c) was 6.8 (range, 5.7–8.2) (Table 1). Participant recruitment and detailed inclusion/exclusion criteria for both diabetic participants without DR and control participants were also consistent with those previously described in our prior study [15]. Briefly, inclusion/exclusion criteria for diabetic participants without DR involved controlled hypertension (<140/90 mmHg), HbA1c ≤ 10 and the absence of active ocular disease or prior retinopathy treatments, while control participants were normal older adults free of significant ocular or systemic conditions (e.g., retinal vascular diseases, DM, uncontrolled arterial hypertension). All participants had best-corrected visual acuity (BCVA) of 0.30 logMAR (6/12) or better, a refractive spherical equivalent within ±3.00 dioptre (D) and an axial length between 22 and 24 mm (mean = 23.7 mm). This axial length range was selected to limit rather than eliminate variability in retinal magnification based on standard axial length assumptions [25].

**Table 1:** Demographics of participants by group.

Characteristics	Control	Diabetics without diabetic retinopathy	*p*-value
Participants (number of eyes)	31 (46 eyes)	28 (45 eyes)	–
Age (y), mean ± SD (range)	59 ± 7.4 (46–78)	59 ± 7.6 (40–71)	0.77^a^
Gender (Male/Female)	10/21	8/20	0.76^b^
HbA1c (mmol/mol), mean ± SD (range)	-	6.8 ± 0.8 (5.7–8.2)	–
Axial length (mm), mean ± SD	23.6 ± 0.95	23.8 ± 0.90	0.96^c^

*HbA1c* Glycated haemoglobin.

^a^Student’s *t*-test.

^b^Chi-square test.

^c^Linear mixed model.

### Study Procedures

Upon enrolment, participants underwent a standardised clinical protocol. This commenced with comprehensive ophthalmic examinations, including BCVA measured using ETDRS charts, slit-lamp examination and axial length measurements obtained via optical biometry (Zeiss IOLMaster 500, zeiss.com) [15]. Following a 15 min wait time for pupil dilation following the instillation of two drops of tropicamide (Mydriacyl 1%; Alcon Laboratories, Inc., myalcon.com), participants underwent full-field ERG testing in both eyes for retinal function assessment. After 10 min of light adaptation in standard room lighting, PhNR and light-adapted (LA) flash ERGs were recorded. Following the LA ERGs, participants underwent 20 min of dark adaptation before dark-adapted (DA) flash ERGs were recorded. After the completion of ERG testing, participants underwent colour fundus imaging. As described previously [15], ultra-wide field images (133° image with 7 μm resolution, Zeiss CLARUS 500, zeiss.com) were acquired and montaged to achieve a 200° field of view. Two masked, experienced graders (AR, KB) classified diabetic participants independently according to the ETDRS protocol and the proposed new international classification, defining the absence of DR by the lack of retinal vascular abnormalities [26, 27], with discrepancies being resolved through discussion. Colour fundus images of the controls were also graded to make sure they met the inclusion and exclusion criteria. Finally, spectral domain OCT (SD-OCT) and OCTA imaging were performed. Detailed descriptions for each assessment are provided in their respective sections below.

### Structural Assessment of the Retina

**Fig. 1:**
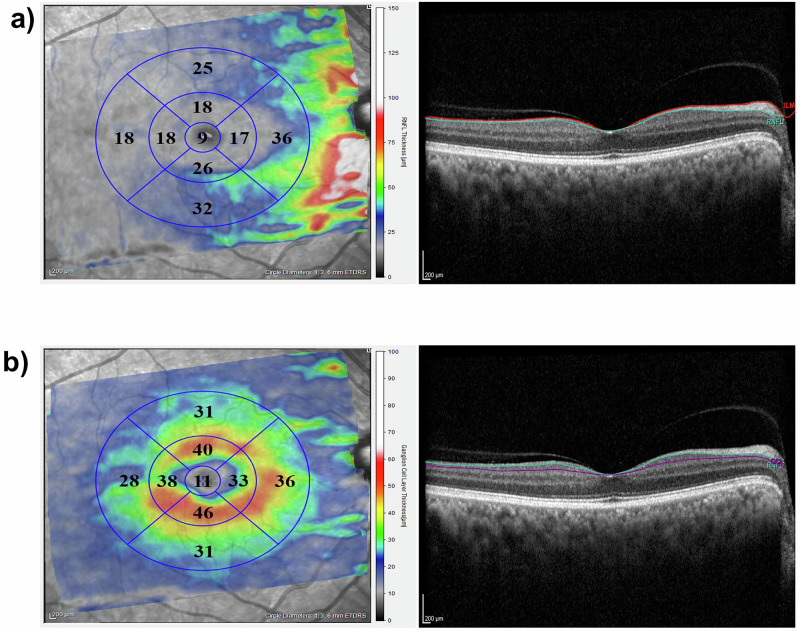
Retinal layer segmentation and thickness measurement using optical coherence tomography (OCT) in a diabetic patient without clinical retinopathy. **a** Retinal nerve fibre layer (RNFL) thickness is shown on a cross-sectional OCT B-scan (right) with an overlaid Early Treatment of Diabetic Retinopathy Study (ETDRS) grid (left) defining subfields for precise RNFL thickness (µm) derivation and **b** ganglion cell layer (GCL) thickness is presented on another cross-sectional OCT B-scan featuring automated GCL segmentation (right) and an ETDRS grid for GCL thickness computation (left).

Retinal structural parameters were assessed using SD-OCT (Spectralis HRA+OCT and Eye Explorer 1.10.4.0; Heidelberg Engineering; heidelbergengineering.com). Macula-centred 30° × 25° OCT scans were acquired for both eyes. The central RNFL and GCL thicknesses were analysed according to the ETDRS protocol. This included measurements from the 1 mm central subfield, 3 mm inner ring (inferior, superior, nasal and temporal quadrants) and 6 mm outer ring (inferior, superior, nasal and temporal quadrants) [26]. These layers were segmented and measured automatically by the Spectralis software. All automated segmentations were visually inspected for accuracy by an experienced grader (SR). The ETDRS grid was automatically centred on the foveal pit, and this centring was confirmed during the quality check. Any scans with significant segmentation errors were manually corrected using the built-in software callipers before the thickness data were exported. For analysis, quadrants were grouped based on their anatomical labels (e.g., ‘inner nasal’ from the right eyes was analysed along with ‘inner nasal’ from the left eyes). As illustrated in Fig. 1a, RNFL thickness was determined from cross-sectional OCT B-scans with an overlaid ETDRS grid, defining subfields for precise thickness derivation. Similarly, Fig. 1b demonstrates the automated GCL segmentation and ETDRS grid application on another cross-sectional OCT B-scan for GCL thickness calculation. The linear (µm) measurements for the ETDRS grid provided by the Spectralis software are based on a standard axial length. Individualised correction for ocular magnification was not applied; however, variability was minimised by restricting the cohort to axial lengths between 22 and 24 mm. The average axial length of the controls (23.6 mm) and diabetics (23.8 mm) did not differ significantly from each other, *p* = 0.96 (Table 1).

### OCTA Acquisition and Analysis

**Fig. 2:**
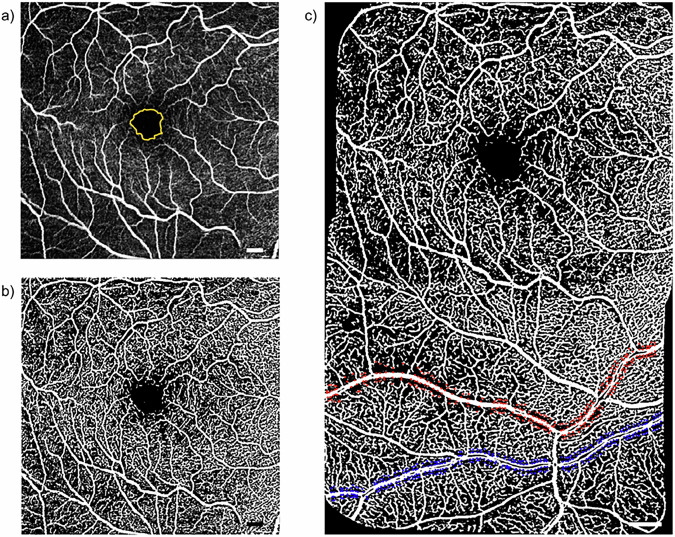
Optical coherence tomography angiography (OCTA) images show vascular parameter measurement workflow in a representative diabetic patient (age 56 years) without clinical retinopathy. **a** A raw macular OCTA image displays the precisely delineated foveal avascular zone (FAZ) for initial assessment (e.g., FAZ area = 0.4 mm^2^; FAZ effective diameter = 713.6 µm). **b** The same macular OCTA image, after processing (e.g., vesselness filtered and thresholded), demonstrates the quantification of the vessel density (vessel density = 0.39), representing the ratio of the image area occupied by retinal vessels (white pixels) to the total area of the image. **c** Illustrates the mid-capillary free zones (CFZs) measurement process on a processed, montaged image. A MATLAB (mathworks.com) script automatically records *x*- and *y*-coordinates belonging to a first-order arteriole (overlaid in red) and a first-order venule (overlaid in blue). The CFZ width is then computed as the Euclidean distance from the edge of a paired first-order arteriole/venule to the middle of the nearest capillary. The middle of the nearest capillary is used because OCTA does not have enough lateral resolution to detect the edge of a capillary. The coloured dots do not represent the CFZ itself, but rather the edge of the paired arteriole or venule and the middle of the nearest capillary from which the Euclidean distances for the CFZs are computed. This analysis of the infero-macular region yielded, for example, a periarteriole CFZ width of 75.7 µm and a perivenule CFZ width of 60.1 µm. Scale bar: 200 µm.

OCTA images were acquired and analysed as described previously in our prior study [15]. Briefly, 20° × 20° OCTA images of paired arterioles, venules and their adjacent capillaries within the macular and infero-macular regions of the superficial vascular plexus were obtained using the Spectralis. This infero-macular region was prioritised for analysis as it consistently provided high-quality, artefact-free images across participants, and represents a mid-peripheral area previously identified as vulnerable to early diabetic changes [15]. Images were montaged using i2k Retina software (DualAlign LLC; dualalign.com), and signal quality was maintained at a minimum of 20. The comprehensive workflow for acquiring and processing OCTA images to quantify vascular parameters is illustrated in Fig. 2. Mid-peripheral CFZ width, vessel diameter, sampling distance, distance from the fovea and vessel density were quantified using previously validated custom MATLAB (R2018b mathworks.com) scripts [28–30]. These computations were done after vesselness filtering [31, 32] and Otsu thresholding [33] were applied to the cropped raw OCTA images. FAZ area was outlined using the vendor software lasso tool on the raw, unprocessed OCTA images. This was carried out prior to the vesselness filtering, as the FAZ boundary is defined by the innermost capillaries, which are clearly visible on the raw scan and could be artificially distorted by the filtering steps. Its effective diameter was calculated as (4 × FAZ area/π)^1/2^ [15, 28–30]. Vessel density was computed as the ratio of vessel-designated white pixel area to the total image area [15, 30]. Data from both eyes were used for analysis if they were of good quality and met the inclusion criteria. Figure 2a represents a raw macula-centred OCTA image with the delineated FAZ area. Following processing (e.g., vesselness filtered and thresholded), the same macula-centred OCTA image (Fig. 2b) was used to quantify the vessel density, representing the proportion of the image area occupied by retinal vessels [15, 30]. Fig. 2c highlights the computation of the mid-peripheral CFZs around the arterioles (indicated by red dots; periarteriole CFZs) and venules (indicated by blue dots; perivenule CFZs) within the superficial vascular plexus for the first-order branches of the vessels. The “mid-peripheral” region in this study refers to the infero-macular area captured by the 20° × 20° OCTA scans, as described in our prior works [15, 28–30]. The CFZ width was calculated using a custom MATLAB script on the processed (filtered and thresholded) images. In brief, this script automatically records *x*- and *y*-coordinates evenly sampled along the edge of a paired first-order arteriole or venule and the middle of the nearest capillary (Fig. 2c) [15, 28–30]. The middle of the nearest capillary was used because OCTA does not have enough lateral resolution to detect the edge of a capillary. The *x*- and *y*-coordinates are then used to compute the Euclidean distance from the edge of an arteriole/venule to the middle of the nearest capillary, termed the periarteriole or perivenule CFZ, respectively [15, 28–30]. This method of computing the mid-peripheral CFZs has been detailed and validated in our prior works [28–30]. As with the structural OCT, linear units for OCTA-derived metrics (e.g., CFZ width in µm, FAZ area in mm²) are based on the device’s standard magnification assumptions and were not individually corrected. However, the strict axial length inclusion criteria minimise this potential source of error. It is also important to note that because vessel density is calculated as a ratio, it is relatively robust to minor variations in retinal magnifications. This effect is further mitigated by the narrow axial length range maintained in this study.

### Functional Assessment of the Retina

Of the 45 eyes from 28 diabetic participants without DR included in the study, 21 eyes from 12 participants successfully underwent ERG testing. The remaining 16 participants (24 eyes) in the diabetic group either declined the ERG procedure (*N* = 12 eyes), could not tolerate the Dawson–Trick–Litzkow (DTL) electrode (*N* = 8 eyes) or had recordings that did not meet quality standards due to excessive artefacts (*N* = 4 eyes). Due to the exploratory nature of the functional assessment in this specific cohort, these metrics are presented as pilot data. To evaluate retinal function, full-field flash ERGs were acquired on an Espion E3 system (V6.64.14, Diagnosys LLC; diagnosysllc.com) following the International Society for Clinical Electrophysiology of Vision (ISCEV) standard for full-field ERGs. The ERG responses were recorded in both eyes after pupillary dilation using a fibre thread (DTL) that rested along the lower lid margin in contact with the inferior cornea as an active electrode. A Grass gold cup electrode (Natus Medical Inc.; natus.com) placed 1 cm lateral to the outer canthus served as the reference electrode, while the ground gold cup electrode was placed at the centre of the forehead. Before the ERG tests began, the surface was cleaned using a skin preparation gel (Nuprep, Weaver and Company; weaverandcompany.com) prior to placing the electrodes on the skin surface using a conductive paste (Ten20, Weaver and Company; weaverandcompany.com) to ensure low impedance.

During ERGs, retinal stimulation was achieved using a Ganzfeld stimulator (ColorDome, Diagnosys LLC; diagnosysllc.com). The ERG protocol began with the LA PhNR, flash and flicker ERGs, followed by the DA flash ERGs. The PhNR stimuli were 4 Hz and 1 Hz red flashes of strength 2 cd.s.m^−2^ against a constant blue background of 10 cd.m^−2^. For each stimulus frequency, responses were averaged across 25 sweeps per repetition for a total of four repetitions and quantified in terms of PhNR amplitude and peak time. The LA standard flash (3.0) ERGs were recorded with a white flash of strength 3 cd.s.m^−2^ and frequency of 1 Hz against a constant white background of 30 cd.m^−2^. The stimuli for LA flicker ERGs were 30 Hz white flashes of strength 3 cd.s.m^−2^ superimposed on a constant white background of 30 cd.m^−2^. The DA ERGs were recorded using white flashes of varying strengths—dim (0.01 cd.s.m^−2^), standard (3 cd.s.m^−2^) and strong (10 cd.s.m^−2^)—on a constant dark background (0 cd.m^−2^). The LA and DA flash ERG responses were averaged over 3–5 sweeps, amplified (10,000×) and quantified in terms of amplitudes and peak times for a-wave and b-wave components.

### Statistical Analyses

All statistical analyses were conducted using R version 4.4.1 (R Foundation for Statistical Computing; r-project.org) with the tidyverse, lmerTest and pROC packages. Results are presented as mean ± standard deviation (SD). Normality of the data was assessed by examining distributions for all outcome variables within each group (control and diabetic). Given that kurtosis and skewness values were within ±3.50 for all variables and considering the total sample size (*N* = 91 eyes), parametric tests were used as per established guidelines [15].

**Fig. 3:**
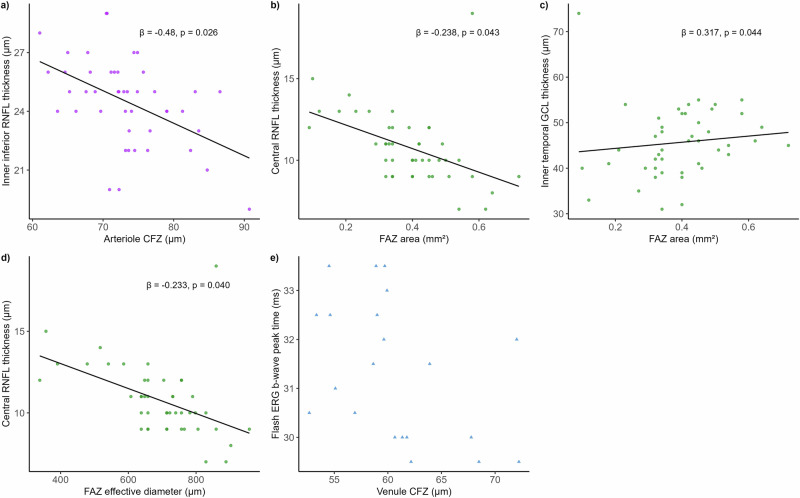
Correlations between vascular parameters and retinal structure/function in diabetic patients without diabetic retinopathy (DR). Standardised β coefficients and uncorrected *p*-values are derived from linear mixed-effects models. These relationships highlight associations between vascular and structural parameters in diabetic participants without DR. **a** Periarteriole capillary free zones (CFZs) versus inner inferior retinal nerve fibre layer (RNFL) thickness. This scatter plot depicts a significant negative association (*β* = −0.48, *p* = 0.03) where wider periarteriole CFZs are related to thinner inner inferior RNFL thickness (μm). **b** Foveal avascular zone (FAZ) area versus central RNFL thickness. This scatter plot illustrates a significant negative association (*β* = −0.24, *p* = 0.04) where a larger FAZ area is associated with thinner central RNFL thickness. **c** FAZ area versus inner temporal ganglion cell layer (GCL) thickness. This scatter plot shows a significant positive association (*β* = 0.32, *p* = 0.04) where a larger FAZ area is associated with thicker inner temporal GCL thickness. **d** FAZ effective diameter versus central RNFL thickness. This scatter plot demonstrates a significant negative association (*β* = −0.23, *p* = 0.04) where a larger FAZ effective diameter is associated with thinner central RNFL thickness. **e** Perivenule CFZs versus flash electroretinography (ERG) b-wave peak time. This scatter plot shows the non-significant relationship between the two parameters (*β* = −0.38, *p* = 0.10).

Linear mixed-effects models (LMMs) were employed to assess differences in vascular and structural parameters between controls and diabetic participants without DR, and to evaluate associations between vascular predictors and structural/functional outcomes within the diabetic participants without DR group. For all LMMs, groups or the vascular predictor were treated as a fixed effect and a random intercept (random effect) for each participant was included. This approach (random effect; using the formula … + (1 | patient_id)) accounts statistically for the non-independence of measurements from fellow eyes by modelling a shared, subject-specific effect. For the comparison of vascular and structural parameters between the control and diabetic groups (Table 2), *p*-values from the LMMs were adjusted for multiple comparisons using the Bonferroni correction. For the association models (Fig. 3), exploring relationships within the diabetic participants without DR group, *p*-values were uncorrected as these represented planned comparisons of specific hypothesised relationships.

**Table 2:** Comparison of vascular, structural and functional parameters between groups using linear mixed-effects models.

		Mean ± SD		
Domain	Variable	Control	Diabetics without diabetic retinopathy	*p*-value^b^
Vascular	Arteriole CFZs (µm)	67.3 ± 7.08	73.4 ± 6.49	**<0.001**
	Arteriole diameter (µm)	92.8 ± 10.4	93.9 ± 12.0	0.46
	Arteriole distance from fovea (°)	13.0 ± 2.61	14.1 ± 2.80	0.07
	Arteriole sampling distance (mm)	6.02 ± 1.28	5.74 ± 0.97	0.34
	FAZ area (mm²)	0.317 ± 0.121	0.391 ± 0.137	**0.02**
	FAZ effective diameter (µm)	623 ± 127	693 ± 134	**0.03**
	Venule CFZs (µm)	54.9 ± 4.58	61.0 ± 6.37	**<0.001**
	Venule diameter (µm)	116 ± 14.5	118 ± 15.2	0.54
	Venule distance from fovea (°)	14.8 ± 1.55	15.4 ± 1.85	0.20
	Venule sampling distance (mm)	5.90 ± 1.02	5.69 ± 0.840	0.32
	Vessel density	0.508 ± 0.032	0.487 ± 0.044	**0.045**
Structural (RNFL)	Central RNFL thickness (µm)	11.9 ± 2.01	10.8 ± 2.15	0.05
	Inner inferior RNFL thickness (µm)	24.9 ± 2.67	24.5 ± 2.23	0.38
	Inner nasal RNFL thickness (µm)	20.4 ± 1.71	19.7 ± 2.89	0.14
	Inner superior RNFL thickness (µm)	23.6 ± 2.65	23.0 ± 3.40	0.35
	Inner temporal RNFL thickness (µm)	16.9 ± 1.21	17.3 ± 1.68	0.19
	Outer inferior RNFL thickness (µm)	39.0 ± 4.00	36.8 ± 5.70	**0.047**
	Outer nasal RNFL thickness (µm)	47.0 ± 6.01	44.7 ± 6.65	0.08
	Outer superior RNFL thickness (µm)	36.7 ± 4.42	36.7 ± 6.25	0.93
	Outer temporal RNFL thickness (µm)	18.7 ± 1.31	18.8 ± 1.73	0.83
Structural (GCL)	Central GCL thickness (µm)	13.8 ± 3.34	12.6 ± 4.69	0.17
	Inner inferior GCL thickness (µm)	51.9 ± 3.47	50.0 ± 7.00	0.11
	Inner nasal GCL thickness (µm)	50.1 ± 4.63	47.6 ± 7.35	0.05
	Inner superior GCL thickness (µm)	51.9 ± 4.09	50.0 ± 6.48	0.08
	Inner temporal GCL thickness (µm)	48.1 ± 3.74	45.6 ± 7.69	0.06
	Outer inferior GCL thickness (µm)	32.7 ± 2.54	33.2 ± 5.08	0.65
	Outer nasal GCL thickness (µm)	38.4 ± 2.92	38.6 ± 4.84	0.99
	Outer superior GCL thickness (µm)	34.6 ± 2.65	34.8 ± 4.26	0.91
	Outer temporal GCL thickness (µm)	34.2 ± 3.57	34.1 ± 5.59	0.85
Functional (ERG)^a^	Dark-adapted ERG a-wave amplitude (µV)		−234 ± 88.7	
	Dark-adapted ERG a-wave implicit time (ms)		14.6 ± 0.947	
	Dark-adapted ERG b-wave amplitude (µV)		297 ± 106	
	Dark-adapted ERG b-wave implicit time (ms)		58.7 ± 10.8	
	ERG a-wave amplitude (µV)		−24.3 ± 65.6	
	ERG a-wave implicit time (ms)		42.5 ± 3.76	
	ERG b-wave amplitude (µV)		204 ± 83.6	
	ERG b-wave implicit time (ms)		99.2 ± 7.05	
	Photopic ERG a-wave amplitude (µV)		−217 ± 99.1	
	Photopic ERG a-wave implicit time (ms)		20.2 ± 3.09	
	Photopic ERG b-wave amplitude (µV)		283 ± 101	
	Photopic ERG b-wave implicit time (ms)		56.0 ± 7.34	
	Photopic ERG Op1 amplitude (µV)		15.4 ± 3.81	
	Photopic ERG Op1 implicit time (ms)		18.5 ± 0.75	
	Photopic ERG Op2 amplitude (µV)		18.6 ± 6.75	
	Photopic ERG Op2 implicit time (ms)		25.3 ± 1.11	
	Photopic ERG Op3 amplitude (µV)		8.49 ± 3.59	
	Photopic ERG Op3 implicit time (ms)		32.1 ± 1.90	
	Flash ERG a-wave amplitude (µV)		−26.9 ± 21.6	
	Flash ERG a-wave implicit time (ms)		15.3 ± 0.80	
	Flash ERG b-wave amplitude (µV)		106 ± 25.3	
	Flash ERG b-wave implicit time (ms)		31.4 ± 1.42	
	Flicker ERG peak amplitude (µV)		76.0 ± 16.5	
	Flicker ERG peak time (ms)		28.3 ± 1.52	
	Flicker ERG trough time (ms)		13.2 ± 1.54	
	1 Hz PhNR a-wave amplitude (µV)		−12.4 ± 6.67	
	1 Hz PhNR a-wave implicit time (ms)		18.6 ± 0.654	
	1 Hz PhNR b-wave amplitude (µV)		69.8 ± 20.8	
	1 Hz PhNR b-wave implicit time (ms)		36.2 ± 1.22	
	1 Hz PhNR-b amplitude (µV)		−16.8 ± 24.3	
	1 Hz PhNR-b implicit time (ms)		73.3 ± 3.67	
	1 Hz PhNR ratio base amplitude		0.365 ± 0.26	
	4 Hz PhNR a-wave amplitude (µV)		−21.0 ± 9.77	
	4 Hz PhNR a-wave implicit time (ms)		18.4 ± 0.516	
	4 Hz PhNR b-wave amplitude (µV)		83.3 ± 21.8	
	4 Hz PhNR b-wave implicit time (ms)		37.1 ± 0.98	
	4 Hz PhNR-b amplitude (µV)		−16.8 ± 24.3	
	4 Hz PhNR-b implicit time (ms)		73.3 ± 3.67	
	4 Hz PhNR ratio base amplitude		0.365 ± 0.26	

Underline – significant or near significant structural parameters included in the combined vascular and structural multimodal receiver operating characteristic (ROC) model.

*CFZs* capillary free zones, ° degrees, *ERG* electroretinography, *FAZ* foveal avascular zone, *GCL* Ganglion cell layer, *µm* micrometres, *µV* microvolts, *mm* millimetres, *ms* milliseconds, *Op1* oscillatory potential 1, *Op2* oscillatory potential 2, *Op3* oscillatory potential 3, *PhNR* photopic negative response, *RNFL* retinal nerve fibre layer, *SD* standard deviation.

^a^Functional testing was only performed in diabetics without diabetic retinopathy group.

^b^Bold values indicate statistical significance at *p* < 0.05; *p*-values are from linear mixed-effects models and were adjusted for multiple comparisons using Bonferroni correction.

Receiver operating characteristic (ROC) analysis was used to compare the discriminative ability of individual parameters and multimodal models. Predictive probabilities for combined models were obtained from generalised linear models with a binomial family and logit link. The ‘Combined Vascular Model’ included periarteriole CFZ, perivenule CFZ, FAZ area, FAZ effective diameter and vessel density. For the ‘Combined Vascular and Structural Model’, all parameters of the vascular model and significant or near-significant structural parameters were considered (Table 2). For each ROC curve, the *p-*value was calculated using the Wilcoxon–Mann–Whitney test, comparing the distributions of the predictor values between the control and diabetic participants without DR groups. The area under the curve (AUC), 95% confidence interval (95% CI), Youden’s Index, optimal cutoff, sensitivity and specificity were reported. In this context, ‘discriminative ability’ refers to the model’s or parameter’s ability to discriminate between the diabetic and control groups, which was quantified using the AUC. Statistical significance for ROC analyses was set at *p* < 0.05.

## Results

### Evaluation of Vascular and Structural Parameters Between Groups

Using LMMs, comparisons between controls and diabetics without DR groups revealed significant differences in key vascular parameters (Table 2). Diabetic participants without DR showed significantly larger periarteriole CFZ width (73.4 ± 6.49 µm versus 67.3 ± 7.08 µm, *p* < 0.001) and perivenule CFZ width (61 ± 6.37 µm versus 54.9 ± 4.58 µm, *p* < 0.001) compared with controls. Additionally, they had a significantly larger FAZ area (0.39 ± 0.14 mm² versus 0.32 ± 0.12 mm², *p* = 0.02) and lower vessel density (0.49 ± 0.04 vs. 0.51 ± 0.03, *p* = 0.045) compared to age-matched controls.

Among structural parameters, the outer inferior RNFL was significantly thinner in diabetics without DR (36.8 ± 5.70 µm) compared to controls (39 ± 4.00 µm) (*p* = 0.047). Several other structural parameters showed trends toward reduced thickness in diabetic participants without DR, including the central RNFL (*p* = 0.05), outer nasal RNFL (*p* = 0.08), inner nasal GCL (*p* = 0.05), inner superior GCL (*p* = 0.08) and inner temporal GCL thickness (*p* = 0.06) (Table 2). Functional ERG parameters were assessed only in the diabetic participants without DR group.

### Association Between Vascular Parameters and Retinal Structure and Function in Diabetic Participants Without DR

To investigate the relationship between vascular parameters (specifically the mid-peripheral CFZs) and retinal structural and functional parameters in diabetics without DR, LMMs were employed, accounting for the correlation between eyes. There was a significant negative association between periarteriole CFZ width and inner inferior RNFL thickness (standardised *β* = −0.48, *p* = 0.03; Fig. 3a). There were no other significant associations between the mid-peripheral CFZs and other sectors of the ETDRS grid for RNFL and GCL thickness (all, *p* > 0.05). This highlights the inner inferior RNFL as a parameter of particular interest in the context of the observed microvascular state. This relationship between periarteriole CFZ width and inner inferior RNFL thickness is represented visually in Fig. 3a, which depicts a scatter plot illustrating this significant negative association.

Beyond the mid-peripheral CFZ parameters, the LMM analyses also revealed significant associations between the FAZ area and central RNFL thickness (standardised *β* = −0.24, *p* = 0.04; Fig. 3b) and inner temporal GCL thickness (standardised *β* = 0.32, *p* = 0.04; Fig. 3c). Similarly, the FAZ effective diameter showed a significant association with central RNFL thickness (standardised *β* = −0.23, *p* = 0.04; Fig. 3d).

Regarding functional correlations, the LMM showed no significant association between the mid-peripheral CFZs and ERG parameters (all, *p* > 0.05). However, the correlation between the perivenule CFZ width and flash ERG b-wave peak time was notable (standardised *β* = −0.38, *p* = 0.10; Fig. 3e).

### Diagnostic Performance of Individual and Multimodal Models

ROC analysis was used to differentiate between controls and diabetic participants without DR (Table 3). Considered independently, perivenule CFZ width demonstrated the highest discriminative ability, as measured by the AUC (AUC = 0.79; 95% CI, 0.70–0.88), followed by periarteriole CFZ width (AUC = 0.72; 95% CI, 0.62–0.83).

**Table 3:** Receiver operating characteristic analysis of individual and combined models for discriminating between control and diabetics without diabetic retinopathy groups.

Parameter/Model	AUC (95% CI)	Youden’s index	Optimal cutoff	Sensitivity	Specificity	*p*-value^a^
Central RNFL thickness	0.675 (0.563–0.786)	0.341	11.5	0.689	0.652	**0.004**
Outer nasal RNFL thickness	0.617 (0.501–0.733)	0.207	43.5	0.511	0.696	0.05
Outer inferior RNFL thickness	0.603 (0.485–0.721)	0.202	34.5	0.289	0.913	0.09
Inner superior GCL thickness	0.585 (0.463–0.707)	0.270	47.5	0.422	0.848	0.16
Inner nasal GCL thickness	0.599 (0.479–0.719)	0.290	43.5	0.356	0.935	0.10
Inner temporal GCL thickness	0.633 (0.512–0.753)	0.358	44.5	0.467	0.891	**0.03**
Arteriole CFZ width (µm)	0.722 (0.618–0.826)	0.364	70.4	0.733	0.630	**<0.001**
Venule CFZ width (µm)	0.791 (0.701–0.882)	0.447	59.5	0.556	0.891	**<0.001**
FAZ area (mm²)	0.657 (0.544–0.771)	0.344	0.285	0.844	0.500	**0.01**
FAZ effective diameter (µm)	0.657 (0.544–0.771)	0.344	602	0.844	0.500	**0.01**
Vessel density	0.660 (0.546–0.773)	0.319	0.509	0.667	0.652	**0.009**
Combined vascular model (CFZs, FAZ, vessel density)	0.828 (0.746–0.909)	0.499	0.278	0.956	0.543	**<0.001**
Combined vascular + structural model	0.854 (0.780–0.928)	0.541	0.350	0.889	0.652	**<0.001**

The combined vascular model includes arteriole CFZs, Venule CFZs, FAZ area, FAZ effective diameter and vessel density.

The combined vascular + structural model includes all parameters from the combined vascular model, plus: central RNFL thickness, outer nasal RNFL thickness, outer inferior RNFL thickness, inner superior GCL thickness, inner nasal GCL thickness and inner temporal GCL thickness.

*AUC* Area under the curve, *CI* Confidence interval, *CFZs* Capillary free zones, *FAZ* Foveal avascular zone, *GCL* Ganglion cell layer, *RNFL* Retinal nerve fibre layer, *µm* micrometres, *mm* millimetres.

^a^*p*-value from Wilcoxon–Mann–Whitney test comparing parameter values (or model probabilities) between control and diabetics without diabetic retinopathy groups; Bold values indicate statistical significance (*p* < 0.05).

**Fig. 4:**
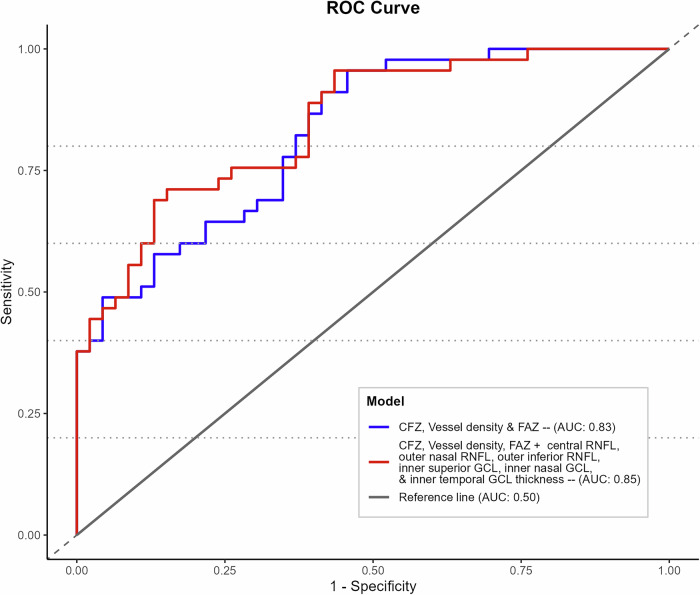
Receiver operating characteristic (ROC) curves for discriminating between controls and diabetic patients without diabetic retinopathy (DR). ROC curves illustrate the diagnostic performance of individual and combined models in differentiating between normal older adults and those with diabetes but no diabetic retinopathy (DR). The discriminatory ability is quantified by the area under the curve (AUC). The diagonal line (AUC = 0.50) serves as a reference, representing a non-discriminatory classifier. The blue line represents the ‘combined vascular model’ (AUC = 0.83, specificity of 0.54, *p* < 0.001), incorporating periarteriole capillary free zones (CFZs), perivenule CFZs, foveal avascular zone (FAZ) area, FAZ effective diameter and vessel density. The red line represents the ‘combined vascular + structural model’ (AUC = 0.85; 95% CI, 0.78–0.93, specificity of 0.65, *p* < 0.001). This expanded model includes all parameters from the combined vascular model, along with significant or near-significant structural optical coherence tomography (OCT) parameters: central retinal nerve fibre layer (RNFL) thickness, outer nasal RNFL thickness, outer inferior RNFL thickness, inner superior ganglion cell layer (GCL) thickness, inner nasal GCL thickness and inner temporal GCL thickness. The inclusion of these structural metrics demonstrates a modest improvement in diagnostic performance.

A combined model of all measured vascular parameters (periarteriole and perivenule CFZs, FAZ area and effective diameter, as well as vessel density) demonstrated a modest discriminative ability (AUC = 0.83; 95% CI, 0.75–0.91) (Fig. 4) with sensitivity and specificity of 0.96 and 0.54, respectively. Adding significant and near-significant structural parameters such as central RNFL, outer nasal RNFL, outer inferior RNFL, inner superior GCL, inner nasal GCL and inner temporal GCL thickness to the vascular model yielded a modestly improved diagnostic performance (AUC = 0.85; 95% CI, 0.78–0.93) and specificity of 0.65, suggesting a marginal improvement in distinguishing diabetic eyes without DR from normal older adults (Table 3, and Fig. 4).

## Discussion

The present study sought to investigate potential associations between the mid-peripheral CFZs versus retinal structure and function in diabetics without DR. The findings suggest the retinal mid-peripheral CFZs are a relevant indicator of this damage. Building upon our previous vascular findings in this cohort [15], this study found a significant association between subtle microvascular pathology and the initial stages of neural degeneration. A key finding was a significant correlation between larger periarteriole CFZ width and a thinner inner inferior RNFL (*β* = −0.48, *p* = 0.03) in the diabetics without DR group. The multimodal model of vascular and structural parameters had a modestly improved AUC and specificity compared with the model of vascular parameters alone (AUC = 0.85 versus 0.83, specificity = 0.65 versus 0.54, respectively). No significant correlation was found between perivenule CFZ width versus flash ERG b-wave peak time (*β* = −0.38, *p* = 0.10). Additionally, a larger FAZ area and effective diameter were significantly associated with a thinner central RNFL but, interestingly, a thicker inner temporal GCL (*β* = 0.32, *p* = 0.04 for FAZ area) in the LMMs.

As indicated earlier, the retinal mid-peripheral CFZs represent the maximum distance that oxygen and nutrients must diffuse to reach the inner retinal neurons, specifically the RNFL and GCL [15, 28–30]. Thus, a larger mid-peripheral CFZ width means that oxygen and nutrients must diffuse across larger distances to reach the RNFL and GCL, leading to potential ischaemia and a defect in the structure and function of these neurons. The significant association of larger periarteriole CFZ with thinner inner inferior RNFL thickness is consistent with this hypothesis. The mid-peripheral CFZs were computed from infero-macular OCTA images; hence, investigating localised association with neurodegeneration, as performed with the ETDRS grids, is imperative. Therefore, it is not surprising that the inner inferior RNFL thickness was significantly negatively associated with the periarteriole CFZ in the LMMs, compared with the other sectors of the ETDRS grids.

Various studies have indicated that neurophysiological and neuropathological changes occur very early in diabetes, even before clinically detectable retinopathy, challenging the idea that primary capillary failure or proliferation initiates the condition [34–36]. Sohn et al. presented evidence that retinal neurodegeneration is not ischaemic in origin, and is primarily related to the duration of diabetes, rather than being a consequence of vascular insufficiency [37]. However, the present findings suggest a more intertwined relationship. The significant link between the mid-peripheral CFZs –a direct measure of microvascular integrity and thinner neural layers like the RNFL support a model where neurovascular decline occurs in concert. This aligns with the concept of the ‘neurovascular unit,’ where neuronal, glial and vascular cells are functionally interdependent, and damage to one component invariably affects the others [38, 39]. The mid-periphery may be a particularly vulnerable region, possibly due to unique metabolic demands or blood flow characteristics, making it an ideal location to detect the earliest signs of diabetic damage [40]. It is important to mention that whether changes in the retinal mid-peripheral CFZs precede changes in RNFL/GCL thickness is a question that can be answered via a longitudinal study design, unlike the present investigation. However, these current cross-sectional results provide a strong foundation to investigate whether changes in the retinal mid-peripheral CFZs are associated with changes in RNFL/GCL thickness via a longitudinal study design.

More recently, Zerbini et al. confirmed that RNFL/GCL thinning precedes retinal ganglion cell death in Akita mice, which is a genetic model for Type I DM, highlighting its potential as an early biomarker and pharmacological target [41]. These collective findings underscore that retinal neurodegeneration is an early event in diabetes, often preceding clinical vascular signs [36]. The present study found significantly thinner outer inferior RNFL thickness as well as trends towards thinner RNFL and GCL in other ETDRS sectors in diabetics without DR, compared with controls. While this study focused on the structural integrity of GCL and RNFL, other researchers have explored the interplay with retinal vasculature. Ki-Young et al. found lower parafoveal vascular density in diabetic eyes without DR, and their longitudinal study suggested that GCL thinning might occur before microvascular changes [42]. Our work, while cross-sectional, also showed a significant association between FAZ metrics and thinner central RNFL in the LMMs beyond the associations shown for the mid-peripheral CFZs. Interestingly, the larger FAZ area was associated with thicker inner temporal GCL thickness. This observed greater thickness of the inner temporal GCL in the presence of a large FAZ area may indicate initial oedema, because of ischaemia caused by reduced blood supply from the perifoveal vessels [43]. In contrast, few studies have shown different results for diabetic patients, particularly those with mild non-proliferative DR (NPDR), compared with those without DR. Retinal thickness measures were greater in the NPDR group in several central, inner and outer ETDRS zones [44]. Also, the overall RNFL and macular ganglion cell/inner plexiform layer (mGCIPL) thicknesses were not significantly different between the no DR and NPDR groups [44]. This suggests that thickness changes might not always be straightforward indicators across all stages or comparisons. Furthermore, Srinivasan et al., in their longitudinal study, also supported this nuance [45]. They found no significant difference in baseline OCT-derived RNFL and mGCIPL thickness in eyes that went on to experience DR progression compared to those with stable DR [45].

Turning to the functional measurements, the current study found a negative correlation between perivenule CFZs and flash ERG b-wave peak time in the diabetics without DR group in the LMMs. Considering the direction of the correlation alone, a reduced flash ERG b-wave in the presence of wider perivenule CFZ width indicates the signal generated by the bipolar and Müller cells in the retina is reaching its peak amplitude (the highest point of the b-wave) faster than normal, but this association was not significant. It is also imperative to mention that measures of full-field flash ERGs provide global rather than localised function, and may account for the lack of significant associations between the mid-peripheral CFZs and ERG parameters. This is because the mid-peripheral CFZs are measures of localised retinal tissue perfusion. Additional measures of retinal function, including pattern ERG and visual field (microperimetry), may reveal a significant association between localised capillary non-perfusion and neural function. Another reason could also be attributed to the reduced number of diabetic eyes that successfully underwent full-field flash ERG. However, Midena et al. detected a reduction of OPs, which suggests subclinical damage to amacrine cells in diabetic eyes during early phases of retinal involvement. Importantly, they found a correlation between OP changes and OCTA parameters, which confirms a relevant role of neurovascular crosstalk dysfunction in the development of DR [46]. Polat Gultekin et al. explored correlations between OCTA-derived vessel densities and various ERG parameters in diabetics without retinopathy and found a positive correlation between N95 amplitudes in pattern ERG and superficial vessel densities in OCTA [47]. They also found a negative correlation between photopic peak times in flash ERG and choriocapillary vessel densities, and no statistically significant correlation between the sum of OP amplitudes and vessel densities in the capillary layers, nor between all OP components and foveal, parafoveal and perifoveal areas in the superficial, deep and choriocapillary plexuses [47]. Similarly, Zeng et al. analysed functional parameters measured by flicker ERG and their associations with corresponding vessel density quantified by OCTA in patients with NPDR and observed a delayed peak time in association with these vascular changes [48].

The diagnostic performance of the current multimodal model, which combines vascular and structural parameters, is particularly noteworthy. The model incorporating periarteriole and perivenule CFZ width, FAZ parameters and vessel density demonstrated a modest discriminative ability (AUC = 0.83, specificity of 0.54, *p* < 0.001), which improved modestly to an AUC of 0.85 (*p* < 0.001) and specificity of 0.65 with the inclusion of RNFL and GCL thickness data. This underscores the enhanced diagnostic ability gained by integrating both microvascular and neural metrics, providing a more comprehensive assessment of the retina’s health in diabetics without DR than any single parameter alone. This multimodal approach could also lead to more robust and reliable clinical decision-making for patients at risk for DR.

The present study contributes to a larger body of evidence emphasising the interconnectedness of neurodegenerative and vascular changes in early diabetes. The consistent observation across many sources that retinal neurodegeneration occurs early, often preceding clinical vascular signs, provides a strong rationale for early intervention and the search for sensitive biomarkers [38]. While this study highlights the promising role of the mid-peripheral CFZs, certain limitations should be considered. The primary limitation is the cross-sectional design, which establishes strong associations but cannot determine causality definitively, nor the temporal sequence of events. Furthermore, the cohort, while well-characterised, was of a modest size and focused exclusively on patients with diabetes without DR. This limits the understanding of how these microvascular changes evolve across various stages of diabetes, such as mild or moderate non-proliferative disease. The lack of a statistically significant correlation with ERG outcomes in the LMMs may be due to the reduced number of diabetic eyes that successfully underwent ERG, that the observed structural changes may precede functional deficits detectable by this specific method or that more sensitive functional tests are required. Similarly, ERG data were collected only for the diabetic group. The absence of a control group for functional assessment means one cannot determine whether the observed ERG values in the diabetic participants without DR are abnormal relative to the specific instrumentation and cohort, thereby limiting interpretation of the functional data. However, it is important to point out that the goal of the study was to build on our initial multimodal vascular model [15], by incorporating structural data and also investigating the association between the vascular, structural and functional parameters in the diabetes group. This was the reason why ERG was not undertaken for the control group. The next steps of this work, which will involve incorporating functional data into the model, will include ERG for the control participants. A further limitation is the recruitment methodology for the control group. While control participants were recruited from our eye clinics—University of Alabama at Birmingham (UAB) EyeCare, UAB Providing Access to Healthcare Clinic, Retina Consultants of Alabama and UAB School of Optometry Satellite Community Clinics based on patients’ medical history and fundus examinations, they did not undergo HbA1c testing in the laboratory [15]. Given the known prevalence of undiagnosed diabetes within the general population, we cannot definitively exclude the possibility that some control participants may have had subclinical diabetes. This could potentially reduce the magnitude of the true differences observed between the groups. A significant limitation in the functional analysis, as noted, is the mismatch between the global nature of full-field ERG, which samples the entire retina and the localised nature of the present mid-peripheral OCTA measurements. This spatial incongruence likely contributed to the lack of significant correlations. More spatially specific functional tests, such as microperimetry, are necessary in future work to map localised functional deficits directly to the specific areas of capillary dropout being measured. Furthermore, the CFZ analysis was restricted to the infero-macular region. While this region was selected for its imaging consistency and prior evidence [15], the analysis was not extended to other mid-peripheral sectors (e.g., superior, temporal or nasal). Consequently, one cannot exclude the possibility that the nature or strength of the association between the mid-peripheral CFZs and neuroretinal thickness may differ in other retinal quadrants. This focused regional analysis limits the generalisability of the findings to the entire mid-peripheral retinal region. Additionally, the structural and vascular metrics (e.g., retinal thickness in µm, FAZ area in mm² and mid-peripheral CFZs in µm) were based on the OCT device’s standard magnification scaling and were not individually corrected for each participant’s axial length. While the axial length range was limited to 22–24 mm to reduce the magnification effects, it should be acknowledged that other biometric parameters, such as corneal curvature and lenticular power, were not individually accounted for. This represents a limitation in the linear scaling and future work would benefit from full biometric-based correction.

These limitations highlight several crucial directions for future research. Future work should prioritise understanding the longitudinal changes of the mid-peripheral CFZs and their relationship with neuroretinal degeneration. Prospective studies are essential to determine the rate of mid-peripheral CFZ expansion and its predictive value for the onset of clinical DR. Such studies would also clarify the temporal relationship between microvascular remodelling and progressive neurodegeneration across different disease stages, including in patients with mild non-proliferative retinopathy. Moreover, strengthening the microvascular and structural correlation with multimodal imaging as a diagnostic parameter remains a key future direction. This would involve integrating OCTA findings with more sensitive functional assessments, such as microperimetry or pattern ERG, to map localised functional deficits to specific areas of capillary dropout [49]. Ultimately, these investigations would be crucial for validating the mid-peripheral CFZs as a robust biomarker for monitoring disease progression and evaluating the efficacy of neuroprotective therapies in early DR. Future research will also incorporate ERG parameters into the current multimodal model that involves vascular and structural parameters. This will involve taking ERG measures for the control participants as well. The lack of significant functional associations may be attributed to the pilot nature of the ERG component and the resulting limited statistical power.

In conclusion, this study identified the mid-peripheral CFZ as a potential marker of early retinal vascular damage, demonstrated its direct association with neuroretinal structural deficits and established a superior diagnostic performance of a multimodal model that incorporates both vascular and structural parameters in diabetic patients without funduscopic evidence of DR. Future work will involve a longitudinal larger sample size design to investigate the between- and within-subject changes in the mid-peripheral CFZs with disease progression, conversion, structural and functional changes. Such a longitudinal study with a large sample size will also facilitate investigation of the possible effects of biological variables such as age, sex, HbA1c levels and ethnicity on mid-peripheral CFZs in individuals with diabetes without clinical retinopathy.

## Data Availability

The datasets generated during and/or analysed during the current study are available from the corresponding author upon reasonable request.
